# The Extended Simple View of Reading in Adult Learners of Chinese as a Second Language

**DOI:** 10.3389/fpsyg.2022.846967

**Published:** 2022-06-16

**Authors:** Meiling Hao, Xiaoping Fang, Zhenzhen Sun, Youyi Liu

**Affiliations:** ^1^College of Advanced Chinese Training, Beijing Language and Culture University, Beijing, China; ^2^School of Psychology, Beijing Language and Culture University, Beijing, China; ^3^Beijing Yizhuang No.1 Primary School, Beijing, China; ^4^State Key Laboratory of Cognitive Neuroscience and Learning, Faculty of Psychology, Beijing Normal University, Beijing, China

**Keywords:** second language learners, Chinese, reading comprehension, decoding, word segmentation, listening comprehension

## Abstract

The Simple View of Reading (SVR) designates that reading comprehension is the product of decoding and listening comprehension and this conclusion has been supported by studies on school-aged native and nonnative speakers. However, it remains unknown whether SVR can be applied to adult second language (L2) learners. The current study addressed this issue by testing adult learners of Chinese as a second language with various proficiency levels and further extended the model by including word segmentation and word-meaning access, both of which are particularly crucial in reading Chinese. The results showed that listening comprehension only contributed to reading comprehension for the advanced learners, while decoding accuracy predicted reading comprehension regardless of Chinese proficiency. However, the total proportion of variance accounted for was relatively low, especially for the lower proficiency groups. Interestingly, word segmentation and word-meaning access explained a large proportion of the total variance and concomitantly decreased the apparent influence of word decoding. Taken together, these findings highlight that the individual characteristics of a given language can modulate the contributions of decoding and listening comprehension to predicting reading comprehension.

## Introduction

Effectively cultivating reading comprehension and exploring the factors affecting its development are key topics in the domain of second language learning and instruction. However, reading comprehension involves various cognitive processes, including but not limited to visual word recognition, word meaning access, word-to-text integration, and inference (Perfetti and Stafura, 2014). The simple view of reading (hereafter SVR) simplified these subskills into two broader categories—word decoding (*word recognition* according to Hoover and Tunmer, 2018) and listening comprehension (or *language comprehension*) and further proposed that reading comprehension is the product of word decoding and listening comprehension (Gough and Tunmer, 1986). Despite the widespread acceptance of SVR in monolingual studies of different writing systems (see Florit and Cain, 2011; Melby-Lervag and Lervag, 2011, 2014; Jeon and Yamashita, 2014; Tighe and Schatschneider, 2016; Quinn and Wagner, 2018; Peng et al., 2021a), its applicability for second language (L2) learners, especially for adult L2 learners, has received much less attention. A few studies support that the SVR can be applied to bilingual speakers (Hoover and Gough, 1990; Barber et al., 2021) and young L2 learners (Sparks and Patton, 2016; Sparks et al., 2018; Sparks and Luebbers, 2018). However, most studies focused on L2 learners whose native and second languages are both alphabetic (Spanish and English in the abovementioned studies). The present study aimed to extend the scope to the reading of a nonalphabetic L2 in adult learners with alphabetic L1, by testing learners of Chinese as a second language (CSL).

### Monolingual Studies Under the Framework of SVR

Since it was initially developed, the SVR has received empirical support from a number of reading studies of monolingual school-aged children. They have confirmed SVR’s adequacy in explaining reading comprehension because that different competencies in word decoding and listening comprehension are able to explain most of the variance in reading comprehension as the SVR predicts [e.g., Language and Reading Research Consortium (LARRC), 2015; Tobia and Bonifacci, 2015; Foorman et al., 2018]. A recent meta-analysis of 42 studies found that word decoding and listening comprehension explained approximately 60% of the variance in reading comprehension (Hjetland et al., 2020). Several studies even observed that these two components accounted for as much as 90% of variance [Adlof et al., 2006; Language and Reading Research Consortium (LARRC), 2015; Foorman et al., 2018; Lonigan et al., 2018]. For example, with a large sample of English-speaking Grade 1–10 students, Foorman et al. (2018) reported that the variance proportion of reading comprehension explained by the two components was between 68 and 99% for each grade, and even reached above 97% in Grades 4–10. Taken together, these findings suggest that word decoding and listening comprehension are essential and adequate to building reading comprehension and that there is little room for other factors to take effect.

Comparatively, there are relatively few studies in the context of Chinese, and extant studies suggest that decoding and listening comprehension explain less variance in reading comprehension in Chinese than in alphabetic languages. For instance, Joshi et al. (2012) compared the relative contributions of decoding and listening comprehension to reading comprehension across Chinese, English, and Spanish in Grade 2–4 children (Joshi et al., 2012). The results showed that in these three languages, decoding and listening comprehension contributed significantly to reading comprehension, but the amount of explained variance in Chinese (25%–42%) was less than that in English (approximately 50%) or Spanish (approximately 60%). In a longitudinal study following Hong Kong primary school children from Grades 1 to 3, decoding and language comprehension each contained several measurable dimensions, including word reading accuracy and text-reading fluency as decoding measure and expressive vocabulary, word definition, oral narrative story comprehension, and syntactic skills as language comprehension dimensions, yet they accounted for less than 40% of the variance in reading comprehension (Yeung et al., 2016). Given these findings, it is possible that the two components in the SVR do not fully capture the reading of Chinese (see also Joshi et al., 2012). Therefore, it is essential to include other components that are particularly important for Chinese reading.

Another line of research revealed that the relative importance of word decoding and listening comprehension to reading comprehension changes as language proficiency increases. A series of studies have reported that the contribution of word decoding decreases with the gradual maturity of decoding skills, whereas the role of listening comprehension increases (e.g., Catts et al., 2005; Diakidoy et al., 2005; Proctor et al., 2005; Foorman et al., 2018; Lonigan et al., 2018; Kim, 2020). For example, a longitudinal study of American primary school children found that, in the second grade, both word decoding and listening comprehension are strong predictors of reading comprehension, but when these students advanced into the fourth grade, the contribution of word decoding decreased, while that of listening comprehension increased (Kim, 2020). In a cross-sectional study on American Grade 3 through five students, Lonigan et al. (2018) observed that listening comprehension (24%–33%) generally accounted for higher unique variance in reading comprehension than decoding (approximately 10%), especially in Grades 4 and 5, and the role of decoding was significantly larger in Grade 3 than in Grade 5.

Furthermore, the relative contribution of the two components and the length of the time window when decoding plays a role are found to be regulated by the orthographic characteristics of the script. In a meta-analysis of 33 studies from English and other shallower orthographies, Florit and Cain (2011) showed that while language comprehension was more influential than decoding accuracy in shallow orthographies, decoding accuracy played a more important role than language comprehension in the early stage of reading acquisition of deep orthography. Specifically, decoding was found to predict reading comprehension for a more extended time window in an opaque orthography (Florit and Cain, 2011). Indeed, Joshi et al. (2015) found that decoding tends to make a substantial contribution to reading comprehension for a long time in primary school in Hebrew—an example of deep orthography (Joshi et al., 2015). However, the findings from Chinese, an orthography typically recognized as among the deepest, are mixed. A meta-analysis of Chinese children’s reading comprehension reported that the role of character or word decoding in reading comprehension began to weaken between Grades 2 and 3 in primary school (Peng et al., 2021a). Ho and colleagues reported that linguistic comprehension was more influential than decoding in predicting children’s Chinese reading comprehension in Grades 1–3 (Ho et al., 2017). Nevertheless, Joshi et al. (2012) found that the importance of decoding increased from Grade 2 to Grade 4. A recent study even found that the role of decoding can last until middle school (Li et al., 2021).

Regarding the relationship between decoding and listening comprehension in predicting reading comprehension, Gough and Tunmer (1986) proposed a multiplicative, not additive, model, which means that the two components are indispensable, and, on statistical analysis, the interaction of the two constructs can significantly predict part of the variance of reading comprehension over and above the contributions of decoding and language comprehension themselves (Gough and Tunmer, 1986). This hypothesis was first confirmed in Hoover and Gough (1990) where the product of decoding and listening comprehension significantly accounted for an extra 1%–7% of variances in English reading comprehension in Grades 1–4 with Spanish speakers learning to read English. However, subsequent studies from alphabetic languages (e.g., Neuhaus et al., 2006; Conners, 2009; Georgiou et al., 2009) and Chinese (Li et al., 2021) failed to replicate such finding. Hoover and Tunmer (2018) explained that “testing such a difference (between additive and multiplicative model) requires a special population where skills are nonexistent for a substantial number of children in at least one of the components” (p309). In fact, some recent studies including multiple grades found that the multiplicative model fits better in the lower grades, while the additive model fits better in the middle and upper grades (e.g., Kershaw and Schatschneider, 2012; Foorman et al., 2020). Yeung et al. (2016) also observed a weak (approximately 1%) but significant contribution of product in Grade 1, but not in Grade 3, in Hong Kong children. Hence, the relationship between decoding and listening comprehension deserves further study.

### SVR on L2 Reading for Bilingual and Second Language Learners

Although originally tested with a group of Spanish-English bilingual children (Hoover and Gough, 1990), the SVR has not received much attention in bilingual or L2 learners’ reading research until more recent years (Proctor et al., 2005). Studies on bilingual children aimed to contrast the SVR’s predictive power across bilingual children with their monolingual peers and revealed a similar pattern as that of monolingual children in which word decoding and listening comprehension explained the majority of the variance in reading comprehension (e.g., Verhoeven et al., 2019; Barber et al., 2021). For example, a longitudinal study by Hoover and Gough (1990) shows that for Spanish-English bilingual children in Grades 1–4, the two components explained 73%–89% of the variance in their L2 reading comprehension. Barber et al. (2021) also observed that these two core components could explain 88.2 and 73% of variance in reading comprehension in English-monolinguals and English-bilinguals, respectively. In a study on young L2 learners who lived in Hong Kong before age 3 and received preprimary education and then formally learned traditional Chinese and Cantonese as their L2 in primary school, Wong (2017) reported that both word decoding and listening comprehension explained 65%–78% of the total variance.

It seems that the SVR framework has been also successful in explaining L2 reading comprehension ability of both bilingual and young L2 learners as Koda (2007) have asserted, however, attention to older L2 learners (i.e., those who start to learn L2 many years later than their L1) under this framework has been rare. The existing studies were mainly conducted by Sparks and his colleagues (Sparks and Patton, 2016; Sparks et al., 2018; Sparks and Luebbers, 2018) and examined the applicability of SVR for US students who began learning Spanish as their L2 only in high school. Their studies showed that although the cohorts of students performed poorly on L2 listening comprehension, vocabulary and reading comprehension, the SVR was also applicable. For example, using multiple regression analyses on students in high school Spanish courses with between 1- and 3-year’ study, the researchers revealed that each of the two components—Spanish word decoding and Spanish listening comprehension—explained approximately 25%–35% of the variance in Spanish reading comprehension, and the product of them added no additional contribution (Sparks and Patton, 2016).

How does the SVR apply to adult L2 learners then? There is no answer to this question yet. In addition to the fundamental differences between children and adults in cognitive maturity, there are complicating factors that seem to exert different effects among adult and younger L2 readers. One of the most important factors lies in the availability of the oral language to contribute to reading comprehension during the process of learning to read (Nation, 2001). It is easy to understand that listening comprehension plays an important role in reading comprehension among monolingual or early bilingual children. Before formally learning to read, children usually have acquired proficient language comprehension skills, so it is easy to transfer these proficient skills to reading comprehension; however, in adult second language learning, listening comprehension, and reading comprehension are often learned at the same time. Therefore, to what extent their language comprehension skills promote the development of reading comprehension is a question to be explored. This study expands our knowledge beyond Sparks’ L2 studies in four aspects—the age acquisition of L2 (adults vs. adolescents), the typological similarity between L1 and L2 (dramatically different vs. similar), the learning context (immersion vs. nonimmersion in the target language), and the relative contributions of different proficiencies across readers.

### Learning Chinese as L2 in Mainland China and Chinese Characteristics

After the turn of the 21st century, the number of foreign students learning in China has increased rapidly and there is a strong demand for Chinese learning. According to the Ministry of Education of the People’s Republic of China, from 2000 to 2018, the total number of foreign students in China increased from 50,000 to 500,000, a tenfold increase. They came from various countries or regions (196 in 2018) and studied in hundreds of universities or colleges. Most of them majored in the Chinese language, while others who had passed the examination for language proficiency level (*Hanyu Shuiping Kaoshi*, hereafter HSK—the standard test of Chinese Language Proficiency for foreign students) and met the language requirements of the university or college chose other subjects. Although different textbooks are used for the teaching of Chinese across universities or colleges, they follow a common principle—the basic skills of listening, speaking, reading, and writing are heavily emphasized for beginners, whereas knowledge about language and culture are added later. Typically, after 1 year of learning, the students’ overall ability in listening, speaking, reading, and writing should reach a basic level of Chinese that enables them to pursue their study in Chinese colleges.

However, foreign students, especially those from countries that do not use Chinese characters in their daily lives, always encounter many obstacles in learning Chinese, because of the huge differences between Chinese and their mother languages. The Chinese writing system is logographic, and no grapheme-phoneme correspondence is available. In addition to the well-known visual complexity, Chinese characters rarely encode phonetic information reliably. Moreover, there are a large number of homophones in Chinese, of which most are monosyllabic and disyllabic words. According to statistics in Liu et al. (2007), there are 31 homophones, on average, for each monosyllabic Chinese word, and the largest one is “yi4,” with as many as 205 Chinese characters sharing the same pronunciation. Based on more commonly used characters, another database (Sun et al., 2018) reported approximately 7.1 homophones, on average, for each Chinese character. Homophones also broadly exist among disyllabic words—the largest number of words in Chinese. The high frequency of homophones can result in the phenomenon where knowing the phonology is not sufficient to access to the exact meaning of a Chinese words. In contrast, the processing of moving orthography to semantics is more reliable and indispensable to disambiguating possible confusion. Most Chinese characters are pictophonetic compound characters, consisting of a semantic radical and a phonetic radical. The semantic radical 氵, for instance, commonly appears in characters that describe liquids, such as 湖 (“lake”), 海 (“ocean”), or 汤 (“soup”). Hence, learners will perform better on reading comprehension tests if they acquire the correspondence between orthography and semantics.

Another challenge for CSL learners and beginning readers is that Chinese, unlike alphabetic languages with clear spaces to separate words, has no explicit word boundaries. This means that the readers have to figure out the boundary of each word during text reading. The majority of modern Chinese words are composed of two characters, but there also exist a large number of single-character words, three-character words, four-character words, and words with even more characters. Given the uncertainty of the number of characters in a word, word segmentation in Chinese becomes even more challenging. Furthermore, Chinese readers are usually confronted with ambiguity in word segmentation during reading. For example, on seeing the four characters “小心地滑,” readers have to consider the context to determine where the word boundary is. When this phrase appears at the entrance of the skating rink, it should be segmented as “小心地/滑,” which means “skate carefully”; however, when it appears on a floor just mopped, the proper meaning is “please be careful! Wet Floor!” and the optimal segmentation should be “小心/地滑.” To achieve word segmentation in Chinese, the readers need to process characters efficiently, and have a mental lexicon with high-quality word representations and adequate probabilistic knowledge about the likelihood of characters comprising a word and their possible positions (Li et al., 2009; Zang et al., 2016). However, CSL learners and beginning readers usually have poor knowledge or awareness about character properties, word representation, and probabilistic information (see also Yang, 2021). Hence, for nonnative Chinese readers, acquisition of the ability to rapidly segment continuous texts into words for accurate lexical access in ongoing reading is reportedly a long process (Everson and Ke, 1997). Studies have also shown that inserting spaces between words or highlighting word boundaries effectively improve reading efficiency among native young children (Blythe et al., 2012; Pan et al., 2021) and CSL learners (Bai et al., 2010; Gao and Jiang, 2015).

### The Present Study

To summarize, reading comprehension studies conducted under the framework of SVR are mostly on alphabetic languages and mainly focus on monolingual, bilingual, or young L2 learners. The applicability of SVR to adults learning a nonalphabetic L2 remains unknown. The present study aims to fill this gap by examining the role of word decoding and listening comprehension in predicting reading comprehension in adult CSL learners.

If the SVR is applicable to adult CSL learners, we will further test the relative importance of decoding and listening comprehension on reading comprehension at different Chinese proficiency levels, as has been done in monolingual studies (e.g., Lonigan et al., 2018; Kim, 2020). Due to the immaturity of listening comprehension in adult CSL learners, we predicted that the contribution of listening comprehension would only be observed in relatively proficient learners. As in alphabetic studies on children, decoding skills were expected to predict adult CSL learners’ reading comprehension, especially among beginners. The product of word decoding and listening comprehension will also be tested to see if any extra contribution is made, especially to beginning learners.

In addition, based on Chinese monolingual studies that show that relatively lower levels of variance are accounted for (Joshi et al., 2012; Yeung et al., 2016), Joshi’s suggestion to use more variables in addition to word decoding and listening comprehension, and the characteristics of Chinese, we examined whether word segmentation and/or word-meaning access make an additional contribution to reading comprehension over and beyond word decoding and listening comprehension. Given their prominences in Chinese word and text reading, we predicted that both word segmentation and word-meaning access would predict reading comprehension in adult CSL learners.

Although the usefulness of making the word boundaries explicit to Chinese reading fluency and comprehension has been extensively explored (Bai et al., 2010; Shen et al., 2012; Gao and Jiang, 2015; Bassetti and Lu, 2016), only two studies have focused on the relationship between the skill of word segmentation and the ability of reading comprehension among CSL learners (Shen and Jiang, 2013; Yang, 2021). However, the two studies obtained very inconsistent results, possibly due to proficiency differences in participants’ Chinese language or the variables controlled.

We tested the participants’ word-meaning access with a meaning-based written vocabulary test (Nation, 1990). Vocabulary size is considered one of the most important predictors of reading comprehension both in the L1 (Perfetti, 2007) and L2 (e.g., meta-analysis of Jeon and Yamashita, 2014) domains. Oral vocabulary is always tested with child participants and often used as an indicator of language comprehension under the framework of SVR, whereas written vocabulary is tested with adolescent or adults, especially in L2 studies (Jeon and Yamashita, 2014). Several CSL studies have observed that meaning-based Chinese written vocabulary has a strong association with Chinese reading comprehension (Zhang and Yang, 2016; Zhang and Koda, 2018; Zhou, 2022), but these studies did not test this association under the SVR framework.

## Materials and Methods

### Participants

Eighty-two adult CSL learners (age mean: 23.73 years, range: 19–33 years; 43 females) participated in the study. They were learning Chinese at an University in Beijing when the study was conducted. According to self-reports, their native languages were Urdu (25), Nepali (13), Spanish (7), Turkish (6), Bengali (5), Indonesia (4), Arabic (3), English (2), French (2), Portuguese (2), Kirghiz (2), Melayu (2), Persian (1), Croatian (1), Serbian (1), Sinhala (1), Turkoman (1), Hungarian (1), Hindi (1), Sonhay (1), and Uzbek (1). None of them were heritage Chinese learners. All participants had studied Chinese in China for at least 6 months when they were tested. The majority of them had taken the HSK, which grades the attendees’ language proficiency into three stages and six levels, among which Levels 1 and 2 belong to the elementary stage, Levels 3 and 4 to the intermediate, and Levels 5 and 6 to the advanced (Peng et al., 2021b). Referring to both their performance on the HSK and their time spent learning Chinese, we assigned 24 participants who had learned Chinese in China for less than 1 year and had never taken part in the HSK to the elementary stage; 30 participants who had spent 1–3 years on learning Chinese and passed Level 3 or 4 on the HSK were intermediate; and 28 participants who had learned Chinese for more than 3 years and passed HSK Level 5 or 6 were advanced.

### Tasks and Materials

Each participant completed six tasks, four of which tapped on word level processing and the other two on listening comprehension and reading comprehension. Similar to the testing of Chinese children in Joshi et al. (2012), the current study measured both decoding accuracy and fluency. However, we used disyllabic words, whereas they utilized monosyllabic characters as stimuli. To maximize the similarity between listening comprehension and reading comprehension in the tested content when tapping the relationship between the two as proposed by Hoover and Tunmer (2018), the same set of texts and questions was used in both listening and reading comprehension tasks. To reduce the repetition effect, we ensured that there was an interval of 2 months between the two comprehension tests.

#### Decoding Accuracy

Tested words were selected from the Syllabus of the Graded Vocabulary for the HSK (*HSK syllabus* hereafter), which included the words that CSL learners need to master for each of the six levels. The word list included 150 disyllabic words in total, of which the number of words from level 1 to level 6 were 10, 10, 20, 30, 35, and 45, respectively. The words were presented in an array of 15 rows and 10 columns. Participants read each word aloud and were instructed to skip words if they did not know a word or simply say “I do not know.” If a participant misread or skipped 15 words in a row, the test was terminated. The number of words that were correctly pronounced divided by 150 was the indicator of decoding accuracy. The Cronbach’s alpha coefficient for all participants was 0.87.

#### Decoding Fluency

Word reading fluency was measured with the speed reading of familiar words. Another set of 100 disyllabic words from levels 1 to 3 of the *HSK syllabus* were selected. The words were expected to be familiar to the participants, and none were used in the word reading accuracy test. All the words were printed on A4 paper in a 10 × 10 matrix. Participants read each word as quickly and accurately as possible and skipped words they did not know. The number of correctly pronounced words and the time taken to read the words were recorded. The number of words read correctly per second was used to indicate decoding fluency. The Cronbach’s alpha coefficient for all participants was 0.90.

#### Word Segmentation

This task examined the ability to segment Chinese words from word strings, which mimics the real situation of Chinese text reading where no physical boundary between words exists. We adapted the task from Liu et al. (2017). Referring to the *HSK Syllabus* and textbooks participants used, we chose 180 words that were expected to be familiar to the participants, including a mix of single-character words, two-character words, three-character words, and four-character words. Each string consisted of three words and a total of 60 word strings were presented in three columns. Participants segmented words in each string by placing slashes between words as accurately and quickly as possible within 90 s. For example, “今年鸡蛋昨天 (this year egg yesterday)” should be divided into “今年/鸡蛋/昨天” and “没问题走打篮球 (no problem walking playing basketball)” should be divided into “没问题/走/打篮球.” The score for each participant was the number of words correctly segmented per second. The Cronbach’s alpha coefficient for all participants was 0.84.

#### Word-Meaning Access

This test was adapted from Nation (1990) to examine the ability to access word meaning from written words with a matching format. Sixty words were sorted by increasing difficulty level according to the *HSK Syllabus* and were divided into 10 groups, with six words in each group. Three definitions were also provided for each group, which matched three of the six words. The words were numbered and presented in the left column and the definitions were presented in the right column. Participants matched the definitions and words by putting the index number in front of each definition. One correct matching earned one point, so the full score was 30. The ratio of earned scores was calculated for each participant. The Cronbach’s alpha coefficient for all participants was 0.88. For example:

1. 意思 [meaning]
2. 衣服 [clothes]     ____ 可以穿的 [the things that you wear]
3. 非常 [very]          ____ 为什么 [why, how]
4. 高兴 [happy]      ____ 很 [very, quite]
5. 医生 [doctor]
6. 怎么 [how, why]

(Note: the answers are 2, 6, and 3 successively for this group. English translations in the brackets were not provided to participants.)

#### Listening Comprehension

This task examined the participants’ ability to understand passages presented out loud. Six passages with varying difficulty levels from Level 1 to Level 6 of the HSK were selected to construct two versions of materials to fit different levels of CSL learners. The difficulty level of words and sentences in the text was also confirmed with Chi-Editor (Jin et al., 2018), a tool to measure text difficulty for CSL learners. Four passages with difficulty levels of 1–4 (easy version) were used to test the elementary group, whereas four passages with difficulty levels of 3–6 (hard version) were used to test both the intermediate and advanced groups. Hence, two passages from levels 3 and 4 were shared in the two versions. The lengths of the passages ranged from 245 to 368 Chinese characters for the easy version and from 334 to 477 Chinese characters for the hard version. For each version, three passages were narrative, and one was expository. After the presentation of a passage, participants completed four written multiple-choice questions to assess their comprehension. The questions focused on information retrieval, main idea extraction, prediction based on the given information, or information interpretation and integration. Participants’ listening comprehension was indicated by their accuracy in answering the questions. The Cronbach’s alpha coefficients were 0.57 and 0.72 for the easy and hard versions, respectively.

#### Reading Comprehension

The materials and the procedure were the same as in the listening comprehension task, except that the passages were presented visually. The Cronbach’s alpha coefficients were 0.69 and 0.71 for the easy and hard versions, respectively.

## Data Analyses and Results

Data analyses were implemented with R software (R Core Team, 2021), the package “sjplot” (version 2.8.10) was used to print the regression models in tables (Lüdecke, 2021), the package “dominanceanalysis” was used to compare the relative contributions of predictors (Navarrete and Soares, 2020), and the function “step()” of stepwise Akaike Information Criterion (AIC)-based regression was applied to choose the best model (Venables and Ripley, 2002).

### Group Comparisons on Each Task

The descriptive statistics and the results of group comparisons are reported in Table 1. Group differences at the four word-level tasks were tested using one-way ANOVA and *post hoc* pairwise comparisons between groups were adjusted using the Tukey HSD correction. All the ANOVAs on reading accuracy, reading fluency, word segmentation, and meaning access indicated significant “group” effects (all *p*s < 0.001), showing a significant improvement across the three proficiency groups. The detailed comparison between groups is shown in the last column of Table 1. Since a different version of materials was used to assess the elementary group’s listening and reading comprehension, we were not able to directly compare the elementary group and the intermediate or advanced group. Instead, we only compared the differences between the intermediate and advanced groups. The Welch two-sample *t* test indicated that the advanced group performed better than the intermediate group on listening comprehension (*t* = 5.954, *df* = 53.203, *p* < 0.001; 95% CI = [0.164, 0.330]), but not on reading comprehension (*t* = 1.346, *df* = 52.194, *p* = 0.184; 95% CI = [−0.027, 0.139]).

**Table 1:** Descriptive data of variables and the results of group comparisons.

Task	Elementary (*n* = 24)	Intermediate (*n* = 30)	Advanced (*n* = 28)	Group differences
Reading accuracy	0.37 (0.13)	0.62 (0.13)	0.78 (0.14)	A > I > E
Reading fluency	0.85 (0.30)	1.19 (0.34)	1.38 (0.33)	A = I > E
Word segmentation	0.32 (0.09)	0.38 (0.12)	0.49 (0.13)	A > I = E
Meaning access	0.70 (0.17)	0.81 (0.15)	0.92 (0.10)	A > I > E
Listening comprehension	0.42 (0.17)	0.52 (0.18)	0.78 (0.13)	A > I^*^
Reading comprehension	0.69 (0.19)	0.76 (0.18)	0.81 (0.13)	A = I^*^

A—advanced group, I—intemediate group, and E—elementray group; group differences were tested after ANOVAs with Tukey-HSD correction on *p* < 0.05.

* indicates only two groups (intermediate and advanced) could be compared for the two tasks, since elementary group was tested on a different version of material. So here is the *t* test result.

### Correlations Between Variables

The Pearson correlations between the six tasks for each group of CSL learners are presented in Table 2. Decoding accuracy correlated significantly with reading comprehension and stayed at a relatively strong level regardless of Chinese proficiency (*r*s = 0.49, 0.43, and 0.72, respectively), whereas the correlations between decoding fluency and reading comprehension were relatively weak and only significant in the elementary group. In contrast, the correlation between listening and reading comprehension increased as Chinese proficiency increased (from *r* = 0.08 for the elementary group to high 0.70 for the advanced group). The correlations between decoding accuracy and listening comprehension were moderate at three levels (0.40 < *r*s < 0.57), indicating that the two components were not independent from each other. The correlation between decoding accuracy and fluency was significant in all three groups and especially high (*r* = 0.83) in the elementary group. Additionally, reading comprehension was significantly related to both word segmentation and word-meaning access across the three groups (0.43 < *r*s < 0.73).

**Table 2:** Correlation between the six variables for CSL learners with elementary, intermediate, and advanced proficiency.

Variables	Elementary (*n* = 24)					Intermediate (*n* = 30)					Advanced (*n* = 28)				
	2	3	4	5	6	2	3	4	5	6	2	3	4	5	6
1. Decoding accuracy (DA)	0.83^***^	0.33	0.71^***^	0.40^*^	0.49^*^	0.35^*^	0.24	0.53^**^	0.54^**^	0.43^*^	0.42^*^	0.62^***^	0.70^***^	0.57^**^	0.72^***^
2. Decoding fluency (DF)		0.55^**^	0.57^**^	0.40^*^	0.49^*^		0.28	−0.11	0.11	0.11		0.46^*^	0.13	0.11	0.31
3. Word segmentation (WS)			0.31	−0.10	0.47^*^			0.30	0.19	0.43^*^			0.26	0.45^*^	0.71^***^
4. Meaning access (MA)				0.30	0.56^**^				0.22	0.73^***^				0.78^***^	0.55^**^
5. Listening comprehension (LC)					0.08					0.28					0.70^***^
6. Reading comprehension (RC)															

* *p* < 0.05;

** *p* < 0.01;

*** *p* < 0.001.

### Regression Analyses

The results of correlational analyses showed that the relationship between different aspects of word recognition and Chinese reading comprehension showed distinct patterns. To further understand the relative contributions of different aspects of word recognition and listening comprehension in predicting learners’ reading comprehension among the three groups of CSL learners, separate regression analyses were carried out to test whether decoding skills and listening comprehension predict reading comprehension well and whether their relative contributions to reading comprehension vary with the learners’ Chinese proficiency.

Three hierarchical regression analyses were carried out, each for one of the three groups, with reading comprehension scores as dependent variables and decoding skill (accuracy or fluency) and listening comprehension scores as predictors. Listening comprehension was entered into the model (Model 1), then decoding (Model 2). Finally, to further clarify the relationship of these two components, the product between decoding and listening comprehension was added to the model at the last step (Model 3). To prevent multicollinearity caused by high correlation between the interaction term and the two variables, centralization was carried out for both before achieving their product. The results are shown in Table 3.

**Table 3:** Regressions on decoding accuracy, listening comprehension, and their product.

	Elementary						Intermediate						Advanced					
	Model 1		Model 2		Model 3		Model 1		Model 2		Model 3		Model 1		Model 2		Model 3	
Predictors	Estimates	*p*	Estimates	*p*	Estimates	*p*	Estimates	*p*	Estimates	*p*	Estimates	*p*	Estimates	*p*	Estimates	*p*	Estimates	*p*
Intercept	0.68	<0.001	0.68	<0.001	0.68	<0.001	0.75	<0.001	0.75	<0.001	0.74	<0.001	0.81	<0.001	0.81	<0.001	0.81	<0.001
LC	0.09	0.717	−0.15	0.513	−0.15	0.527	0.29	0.130	0.07	0.727	0.07	0.745	0.68	<0.001	0.42	0.007	0.39	0.024
DA			0.78	0.015	0.77	0.019			0.55	0.069	0.53	0.085			0.43	0.003	0.42	0.006
LC × DA					0.11	0.944					1.11	0.534					−0.43	0.657
*R*^2^/*R*^2^ adj.	0.006/−0.039		0.254/0.183		0.254/0.142		0.080/0.047		0.187/0.127		0.200/0.107		0.495/0.475		0.645/0.616		0.648/0.604	

LC, listening comprehension; DA, decoding accuracy. Model 1 includes only listening comprehension, then decoding accuracy entered in model 2, and finally the product of listening comprehension and decoding accuracy entered in model 3.

For the elementary group, Model 1 was not significant [*F*_(1,22)_ < 1], indicating that listening comprehension was not a reliable predictor for reading comprehension (*p* > 0.1); When decoding accuracy was added to Model 2, the model became significant [*F*_(2,21)_ = 3.569, *p* < 0.05]. Decoding accuracy was significant (*p* < 0.05), and the explained variance was 18.3% (adjusted *R*^2^ = 0.183). The unique variance explained by decoding accuracy was 22.2% (which equals 18.3 minus −3.9). However, the interaction between the two components failed to explain a significant additional variance as shown in Model 3 (*p* > 0.1).

For the intermediate group, the picture was similar to that of the elementary group. The role of listening comprehension (Model 1) was not significant [*F*_(1,28)_ = 2.431, *p* > 0.1]. The role of the decoding accuracy (Model 2) was marginally significant (*p* = 0.069), and the explained variance reached 12.7%. The unique variance explained by decoding accuracy was 8% (which equals 12.7 minus 4.7). Similarly, the product of the two components was not significant (*p* > 0.1).

For the advanced CSL learners, a different picture was observed. Listening comprehension was significant (*p* < 0.001) and explained 47.5% of the variance in reading comprehension in Model 1 [*F*_(1,26)_ = 25.44, *p* < 0.001]. After adding decoding accuracy, both were significant (*p*s < 0.01), and they explained 61.6% of the variance in reading comprehension in Model 2 [*F*_(2,25)_ = 22.67, *p* < 0.001]. The unique variance explained by decoding accuracy was 14.1% (which equals 61.6 minus 47.5). Again, the product failed to explain any additional variance in Model 3 (*p* > 0.1).

In summary, we found that decoding accuracy predicted reading comprehension regardless of Chinese proficiency, while listening comprehension was a significant predictor of reading comprehension only when learners reached the advanced level of Chinese proficiency. However, the interaction between decoding accuracy and listening comprehension did not significantly explain the additional variance in reading comprehension in any of the groups, which suggested that an additive model was better than a multiplicative one. Importantly, we observed that the total variance explained by decoding accuracy and listening comprehension for the elementary and intermediate groups was low (both adjusted *R*^2^s < 20%), and a sharp increase was observed in the advanced group (adjusted *R*^2^ = 61.6%).

A similar set of regression analyses were also conducted with decoding fluency, listening comprehension, and their product as predictors (see Table 4). We could see that the result pattern was similar to that on decoding accuracy; the difference was that the contribution of decoding fluency was relatively weak when compared to decoding accuracy, since decoding fluency was only significant in the elementary group (the amount of unique contribution was 22.6%, which was similar to decoding accuracy). Again, the total variance explained was very low for the beginning and intermediate groups (especially low on the intermediate group since neither of the two predictors was significant), and the adjusted *R*^2^ on the advanced group jumped to 51.3%.

**Table 4:** Regressions on decoding fluency, listening comprehension, and their product.

	Elementary						Intermediate						Advanced					
	Model 1		Model 2		Model 3		Model 1		Model 2		Model 3		Model 1		Model 2		Model 3	
Predictors	Estimates	*p*	Estimates	*p*	Estimates	*p*	Estimates	*p*	Estimates	*p*	Estimates	*p*	Estimates	*p*	Estimates	*p*	Estimates	*p*
Intercept	0.68	<0.001	0.68	<0.001	0.68	<0.001	0.75	<0.001	0.75	<0.001	0.74	<0.001	0.81	<0.001	0.81	<0.001	0.81	<0.001
LC	0.09	0.717	−0.16	0.499	−0.15	0.536	0.29	0.130	0.28	0.150	0.25	0.192	0.68	<0.001	0.66	0.007	0.66	0.024
DF			0.35	0.014	0.34	0.019			0.04	0.679	0.10	0.397			0.09	0.094	0.09	0.101
LC × DF					0.07	0.927					−0.73	0.302					−0.05	0.925
*R*^2^/*R*^2^ adj.	0.006/−0.039		0.257/0.187		0.258/0.146		0.080/0.047		0.086/0.018		0.123/0.022		0.495/0.475		0.549/0.513		0.549/0.493	

LC, listening comprehension; DF, decoding fluency. Model 1 includes only listening comprehension, then decoding fluency entered in model 2, and finally the product of listening comprehension and decoding fluency entered in model 3.

In the next sets of analyses, we tested whether word segmentation and word-meaning access make extra contributions to reading comprehension over and above word decoding and listening comprehension.

We first constructed a full model (Model 1) of regression with all five variables (i.e., listening comprehension, decoding accuracy, decoding fluency, and word segmentation, and meaning access) as the predictors of reading comprehension for each group and used the function “step” to detect its best model (Model 2) with the lowest AIC from all possible models. The results are summarized in Table 5.

**Table 5:** Regressions on listening comprehension, decoding accuracy and fluency, word segmentation, and meaning access.

	Elementary				Intermediate				Advanced			
	Model 1		Model 2		Model 1		Model 2		Model 1		Model 2	
Predictors	Estimates	*p*	Estimates	*p*	Estimates	*p*	Estimates	*p*	Estimates	*p*	Estimates	*p*
Intercept	0.58	0.227	0.28	0.187	0.51	0.189	0.45	0.105	0.57	0.135	0.82	0.001
LC	−0.25	0.496			0.21	0.416			0.57	0.022	0.56	0.003
DA	0.28	0.766			−0.52	0.384			0.44	0.309		
DF	0.16	0.706			0.02	0.905			−0.01	0.959		
WS	0.20	0.459			0.25	0.166	0.26	0.114	0.34	0.044	0.43	0.001
MA	0.41	0.259	0.66	0.013	0.84	0.001	0.73	<0.001	−0.13	0.535		
*R*^2^/*R*^2^ adj.	0.371/0.196		0.247/0.213		0.522/0.422		0.500/0.463		0.662/0.585		0.644/0.615	

LC, listening comprehension; DA, decoding accuracy; DF, decoding fluency; WS, word segmentation; and MA, meaning access. Model 1 includes all five predictors, whereas model 2 is the best regression model selected for each proficiency.

For the elementary group, no variable was significant in the full model (all *p*s > 0.1), and only meaning access was a reliable predictor of reading comprehension in the best model (*p* < 0.05). The explained variance was 21.3%, as shown in the value of the adjusted *R*^2^. For the intermediate group, again, only the variable of meaning access was significant in both the full model and the best model (*p*s < 0.001), but the explained variance jumped to 46.3%. A different pattern was observed in the advanced learners; both listening comprehension and word segmentation predicted reading comprehension in the full model (*p*s < 0.05) and in the best model (*p*s < 0.01). The two variables explained 61.5% of the variance in reading comprehension. In short, listening comprehension again predicted reading comprehension only when learners reached a high level of Chinese proficiency. More importantly, word decoding (accuracy or fluency) became no longer significant in any group when word segmentation and meaning access were added to the model, but the explained variance increased, especially in the intermediate group (46.3% vs. 12.7% or 1.8%).

**Figure 1:**
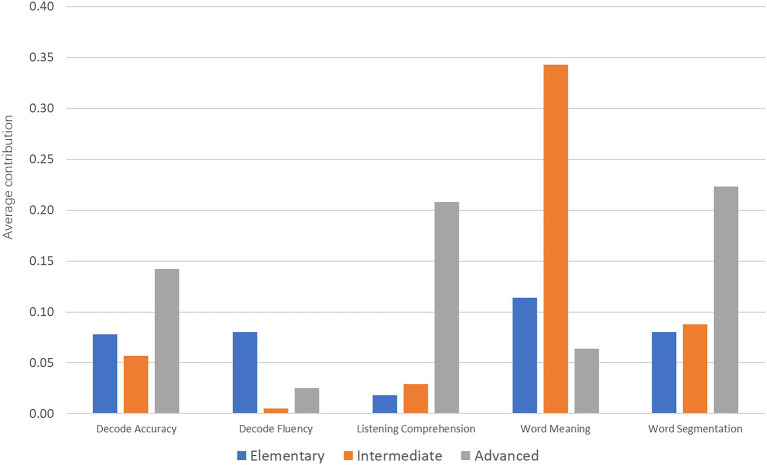
The average contribution of each predictor at the three groups of learners.

Finally, we ran dominance analysis (DA) to directly assess the relative importance of the predictors of reading comprehension in each group. Multiple regression analysis allows researchers to explore relationships between predictors and outcome variable. However, it is difficult to interpret the importance of individual predictors when there is a high degree of multicollinearity between variables. DA is an extension of multiple regression developed by Budescu (1993), and it addresses the issue of highly correlated variables. DA relies on estimating an *R*^2^ value for all possible comparisons of predictors as they relate to a criterion (i.e., reading comprehension here). Among the three types of dominance, general dominance is achieved if a predictor’s additional contribution is greater across the average of all conditional values compared with the competitor predictor. The average contribution, which is calculated by averaging all contributions by possible combinations, as shown in Figure 1, defines general dominance.

For the elementary learners, the contribution of decoding accuracy and fluency, meaning access, and word segmentation was approximately 10% (7.8%–11.4%), and listening comprehension was only 1.8%. For the intermediate learners, coinciding with the above best-model analysis, meaning access turned up a bump contribution (34.3%), whereas the other four were relatively weak (approximately 5%–10% for decoding accuracy and word segmentation and less than 3% for decoding fluency and listening comprehension). For the advanced learners, both listening comprehension and word segmentation had contributions larger than 20% (20.8 and 22.3%, respectively), and decoding accuracy also contributed nearly 15%.

## Discussion

The present study tested whether word decoding and listening comprehension could predict the reading comprehension of adult learners of Chinese as a second language (CSL) under the SVR framework and explored their relative contributions across proficiency levels. We then extended the SVR by including measures of word-meaning access and word segmentation to better capture the characteristics of Chinese reading and examined their contributions over and above word decoding and listening comprehension. We discuss each of the main findings below.

### Word Decoding and Listening Comprehension for Adult CSL Learners

First, we found that decoding accuracy made a significant contribution to reading comprehension across proficiency levels. This finding that decoding plays a role over a long period of time is consistent with what has been found in both native Chinese children (Joshi et al., 2012) and young CSL learners (Wong, 2017, 2019). Furthermore, our finding is in line with that from other deep orthographies, such as Hebrew and English (Joshi et al., 2012; Lonigan et al., 2018). For example, with a similar task to ours in a cross-sectional study, Joshi et al. (2012) observed that decoding accuracy in both the English and Chinese groups accounted for a significant amount of the variance of reading comprehension at both Grade 2 and Grade 4; however, the effect of decoding waned and became nonsignificant at Grade 3 for the Spanish group. After comparing the studies in English and those in other more transparent orthographies, Florit and Cain (2011) made a similar finding in a meta-analysis study, which indicated that decoding accuracy remained a strong influence even for Grade 5 readers in English, but its role in more transparent orthographies was relatively weak and lasted for a shorter period of development. Hence, researchers have proposed that the orthographic depth of the writing system regulates the relationship between decoding and reading comprehension (e.g., Florit and Cain, 2011). The skill of decoding can be acquired by the end of the first instruction year for the children in languages with transparent orthography (like Finnish), but the process in languages with a deep orthography, such as English, progresses at a slower rate (see Seymour et al., 2003). Unlike the alphabetic writing system, there is no grapheme-phoneme correspondence for Chinese characters at all; thus, Chinese native readers or CSL learners need to spend much time practicing to crack the code between orthography and phonology of Chinese characters during reading acquisition.

We also observed a significant contribution of decoding fluency only for the CSL beginning learners (Table 4). This pattern was similar to the finding for Chinese children in Joshi et al. (2012), which showed that decoding fluency had a significant role in Grade 2 but diminished to near zero in Grade 4. However, this pattern of decrease with grade differs from Yeung et al. (2016). In a longitudinal study on Chinese Hong Kong children, Yeung et al. (2016) found that text fluency had a stable contribution to passage reading comprehension in both Grades 1 and 3. We should note that the fluency index is passage text-based in Yeung et al. (2016), whereas that in Joshi et al. (2012) and in the present study is isolated character- or word-based, which may explain why we observed a different pattern from Yeung et al. (2016). Although both accuracy and fluency were recognized in the original SVR model (Hoover and Gough, 1990), most studies of the SVR have measured decoding in terms of accuracy only (Kirby and Savage, 2008), especially in those studies focusing on deep orthography. The results from studies of English are inconclusive about the role of decoding fluency, and a proposal has been made that for readers of English, decoding fluency might play an important role in later grades, as texts become more demanding (Aaron et al., 1999). The role of decoding fluency in Chinese reading comprehension needs further investigation, since few studies have focused on it.

In contrast to decoding fluency, we found that listening comprehension was steadily able to predict reading comprehension only in the advanced learners of CSL, even after including word segmentation and word-meaning access in the analysis, but not in the elementary or intermediate groups. The meta-analysis by Florit and Cain (2011) revealed a general developmental pattern across different orthographies in which the influence of listening comprehension on reading comprehension increased with reading proficiency. The role of listening comprehension increased with reading proficiency in our results, which echoes the general developmental pattern above and the findings in Joshi et al. (2012). It has long been acknowledged that when word reading becomes relatively efficient and automatic, a larger proportion of processing resources can be devoted to higher-level comprehension processes (e.g., Perfetti, 1985; Cunningham et al., 1990). This might explain why, in readers with several years of instruction, listening comprehension becomes a more important predictor of reading comprehension. However, the near zero effects of listening comprehension for both the beginning and intermediate CSL learners should be noted. On the one hand, this outcome reflects the orthographic influence, as has been emphasized in a meta-analysis study, in which decoding was more influential than listening comprehension for beginner readers in languages with a deep orthography, such as English (Florit and Cain, 2011). Readers of Chinese must devote more effort to process word recognition and leave fewer resources for comprehension, as we have discussed above. On the other hand, this outcome might be the result of a lack of language comprehension to help reading. Children typically have well-developed language comprehension skills before learning to read in their native languages, which helps them understand what they are reading even in the early stage of reading acquisition. In contrast, adult learners of CSL are usually exposed to spoken and written inputs around the same time, and their listening comprehension is not necessarily better than their reading comprehension (Koda, 2007, 2008; Melby-Lervag and Lervag, 2014); thus, reading comprehension may not benefit from listening comprehension. Indeed, our elementary and intermediate participants performed worse in the listening comprehension task than the reading comprehension task.

Finally, similar to the findings of the majority of this kind of research, the product of word decoding and listening comprehension in the current study failed to explain any additional variation of reading comprehension for any proficiency level of learners and hence lent support to the additive model rather than the multiplicative model (e.g., Tiu et al., 2003; Neuhaus et al., 2006; Conners, 2009; Georgiou et al., 2009).

### Word Segmentation and Word-Meaning Access in Reading Comprehension

In addition to word decoding and listening comprehension, both word segmentation and meaning access were indeed able to predict reading comprehension of adult CSL learners. Furthermore, their contributions were modulated by language proficiency.

As mentioned in the Introduction, only two studies have investigated the process of word segmentation in the reading comprehension of CSL learners and their findings seemed inconsistent. Shen and Jiang (2013) explored this issue among beginners with approximately 1 year of Chinese course learning. Their results showed that word segmentation did not uniquely predict reading comprehension beyond character reading accuracy and fluency. In contrast, Yang (2021) observed that word segmentation was significant to reading comprehension after controlling for word reading fluency. Two key differences should be noted between the two studies. First, the participants in Yang (2021) were selected from students with 1–3 years of Chinese learning and they hence varied in different L2 proficiency levels, while only beginners were included in Shen and Jiang (2013). Second, Yang (2021) did not consider the influence of word accuracy. After controlling for both word accuracy and fluency as in Shen and Jiang (2013), our study only found an effect of word segmentation on the advanced group, not on the other two groups. This result suggests that the role of word segmentation in reading comprehension would change dynamically over L2 proficiency levels.

According to the interactive processing model proposed by Li et al. (2009), Chinese word segmentation can be modulated by both bottom-up (e.g., matching target items to mental lexicon; the combination of the meaning of constituent characters) and top-down (e.g., context constraints; background information) strategies. Empirically, studies have found that to finish the task of Chinese word segmentation, both beginning and advanced CSL learners predominantly employed bottom-up strategies, matching the target item to their existing mental lexicon, and advanced learners used more top-down contextual information than beginners (Shen, 2008). The differences in strategy might be reflected in the correlation coefficients between word segmentation and reading comprehension (Table 2), which showed that the correlation was higher in the advanced group.

The current study showed that visual word-meaning access significantly contributed to adult CSL learners’ reading comprehension, which is consistent with previous studies (Zhang and Yang, 2016; Zhang and Koda, 2018; Zhou, 2022). By using a similar task with a group of CSL learners with intermediate to advanced proficiency levels, Zhou (2022) found that Chinese word-meaning access had a strong predictive power for their Chinese reading comprehension after controlling for morphological awareness. The task we used tracks meaning retrieval from word orthography, as a type of written vocabulary knowledge. It should be noted that written vocabulary differs from oral vocabulary based on everyday conversation (Nagy and Townsend, 2012). Empirically, Zhang and Koda (2018) distinguished the two types of vocabulary in a study with a group of heritage Chinese language learners and found that both vocabulary types also differed in their roles in reading comprehension, indicating that only written vocabulary had a significant contribution to passage comprehension. They explained that the nature of oral vocabulary is conversational, casual, and informal, whereas reading comprehension typically requires linking visual vocabulary to abstract conceptual representations.

Although we did not find an impact of meaning access on the comprehension of advanced CSL learners, this does not imply that meaning access is not crucial for reading. According to the lexical quality hypothesis (LQH; Perfetti, 2007) and the decoding, vocabulary, and comprehension triangle (DVC; Perfetti, 2010), word meanings are central to both reading comprehension and word identification. Moreover, accessing word meanings from visual form is considered to be, compared to transparent orthographies, even more important and more efficient in Chinese reading based on the orthographic depth hypothesis (Katz and Frost, 1992) and the lexical processing model of Chinese reading (Zhou et al., 1999). On the one hand, we noticed that the correlation between meaning access and listening comprehension was high (*r* = 0.78) in the advanced group; on the other hand, the advanced learners possibly used more contextual information to help segment words, as discussed above (Shen, 2008). Hence, it might be that both listening comprehension and word segmentation obscured the role of word-meaning access in reading comprehension. More data and structural equation modeling (SEM) could provide more direct evidence in the future.

### Chinese Reading Comprehension of Adult CSL Learners

Our results also showed that the role of word decoding was no longer significant once word-meaning access and word segmentation were also considered. This data pattern does not necessarily mean that word decoding has no effect on reading comprehension for adult CSL learners and it can be replaced by other variables (e.g., word-meaning access and word segmentation). Instead, word decoding may have an indirect effect on reading comprehension as the DVC model has indicated (Perfetti, 2010), and we tested this hypothesis with SEM as detailed below.

Due to the small sample, we tentatively tested the hypothesis of indirect effect with the method of partial least squares SEM by using the Smart-PLS software (Ringle et al., 2015). Bias-corrected and accelerated (BCa) bootstrapping with 5,000 subsamples was used to test the significance of the effect and to estimate the confidence interval. The model fit index of SRMR was good according to the criterion of 0.08 (elementary = 0.028, intermediate = 0.056). The results confirmed that word decoding had no direct effect on reading comprehension (*p*s > 0.1), but it showed a significant indirect effect mediated by meaning access on both elementary (*effect* = 0.332, *t* = 2.630, *p* < 0.01, 95% CI_corrected_ = [0.095, 0.598]) and intermediate groups (*effect* = 0.339, *t* = 3.331, *p* < 0.001, 95% CI_corrected_ = [0.152, 0.554]).

Hence, we outline the following trajectory of the development of reading acquisition. At the elementary Chinese level, learners are still exploring effective ways to decipher the mapping between Chinese orthography, phonology, and meaning. Due to the unreliability of the phonological route and learners’ poor listening comprehension, only word recognition based on meaning access has a limited role in reading comprehension; With the accumulation of Chinese characters and the growing awareness of the characteristics of Chinese characters, intermediate CSL learners gradually realize the importance of word-meaning access in Chinese reading, and there is a closer relationship between word-meaning activation and reading comprehension. At the advanced Chinese level, learners’ word reading strategies gradually mature, and word recognition becomes automatic, freeing up cognitive resources for word segmentation, and listening comprehension to play a role in the reading process. Whether such a developmental trajectory is reasonable remains to be confirmed by more experimental studies.

### Implications and Limitations

Reading is a complex activity, especially for adult L2 learners with different orthographic L1 backgrounds. The findings highlight the role of subskills, including word decoding, word-meaning access, word segmentation, and listening comprehension, on CSL learners’ reading comprehension and provide practical implications for the learning and instruction of Chinese as a second language. First, word instruction should emphasize both reading aloud and meaning explanations from visual input to improve CSL learners’ lexical representation and ability to recognize Chinese visual words. According to LQH (Perfetti, 2007), high quality lexical representations allow for fast and accurate visual word recognition, which not only helps learners access the precise meaning of words but also frees up cognitive resources to practice higher-level comprehension skills—prediction, reasoning, integration, reflection, and other abilities- to finally improve their reading comprehension skills. With the improvement of reading comprehension skills, learners have more opportunities to be exposed to reading materials, showing the Matthew effect in the development of reading ability (Stanovich, 1986), and finally improving comprehensive skills in the Chinese second language. Second, to develop the students’ ability in word segmentation, repeated and alternated reading on both spaced and unspaced texts should be used, especially for the beginning learners. The spaced texts serve as a model for word segmentation and reading scaffolds (see Yang, 2021). The students then practiced their own word segmentation during rereading phases. Third, students should be taught discourse skills explicitly to improve their listening comprehension ability. The founders of the SVR believe that if written words are recognized, the processing involved in reading comprehension is the same as that involved in listening comprehension. Therefore, teaching in one modality may transfer to another modality. In fact, a recent study found that there is a two-way mutual promotion between reading comprehension and listening comprehension (Wong, 2019).

Admittedly, this study has some limitations. First, a larger sample size would be helpful to generalize our findings, and a sufficient number of L2 participants with the same L1 background will further purify our findings. Second, a single task was used in each measure, which may result in measurement errors. Future research can consider using latent variables in multiple tasks to reduce measurement errors. Third, although we extended decoding skills to broad word recognition with two other tasks, as proposed by Hoover and Tunmer (2018), we did not measure other skills related to word reading, such as metalinguistic awareness. In previous studies, these skills were found to predict reading comprehension (Tunmer and Chapman, 2012; Dong et al., 2020). This may also be one of the reasons why decoding and listening comprehension in our study have smaller contributions to elementary and intermediate CSL learners’ reading comprehension. Future research can integrate these basic language and cognitive skills into the framework of SVR to verify its applicability. Fourth, although our study carefully selected texts from the standardized Chinese proficiency test, we did not consider the influence of text features. In recent years, some studies have used linear mixed-effects models to investigate the influence of text features and readers’ cognitive skills and their interaction on reading comprehension (Francis et al., 2018; Wang et al., 2021). In future research, text characteristics should be incorporated into the SVR framework to better understand the complexity of reading comprehension (RAND Reading Study Group, 2002).

## Conclusion

The current study found that word decoding and listening comprehension, two core cognitive skills in the framework of SVR, contributed to adult CSL learners’ reading comprehension, with the role of listening comprehension present only for relatively proficient learners. Furthermore, we extended the SVR by demonstrating the roles of word-meaning access and word segmentation in adult CSL learners’ reading comprehension by adding large proportion of explained variance in total and decreasing the contribution of word decoding when they acted as predictors. Taken together, the characteristics of language modulate the contributions of word decoding and listening comprehension to reading comprehension. These findings also have important practical implications for the instruction of second language. Specifically, educational activities designed to promote L2 learners’ reading comprehension should incorporate the cognitive processes that are specific to the target languages, in addition to word reading accuracy and fluency.

## Data Availability Statement

The raw data supporting the conclusions of this article will be made available by the authors, without undue reservation.

## Ethics Statement

The studies involving human participants were reviewed and approved by Beijing Language and Culture University. The patients/participants provided their written informed consent to participate in this study.

## Author Contributions

MH and YL: conception and design of the study. ZS, MH, and YL: acquisition, analysis, and interpretation of data. MH, YL, and XF: drafting. All authors contributed to the article and approved the submitted version.

## Funding

This study was funded by National Social Science Foundation of China (Grant Nos. 21BYY171 and 17ZDA305), National Natural Science Foundation of China (Grant No. 31970977), Science Foundation of Beijing Language and Culture University (supported by “the Fundamental Research Funds for the Central Universities”) (Grant Nos. 19PT01 and 21YBB09), and Open Research Fund of the CAS Key Laboratory of Behavioral Science, Institute of Psychology, China.

## Conflict of Interest

The authors declare that the research was conducted in the absence of any commercial or financial relationships that could be construed as a potential conflict of interest.

## Publisher’s Note

All claims expressed in this article are solely those of the authors and do not necessarily represent those of their affiliated organizations, or those of the publisher, the editors and the reviewers. Any product that may be evaluated in this article, or claim that may be made by its manufacturer, is not guaranteed or endorsed by the publisher.
